# Supplementary material for: Wiedemann–Franz behavior at the Weyl points in compressively strained HgTe
Source: Nat Commun. 2025 Nov 29;16:10813. doi: 10.1038/s41467-025-66909-z (PMC12669628; doi:10.1038/s41467-025-66909-z)
Supplement: Supplementary file 1 — Supplementary Information [file 41467_2025_66909_MOESM1_ESM.pdf]

Supplementary Information: Wiedemann-Franz behavior at the Weyl points in compressively strained HgTe

Abu Alex Aravindnath,[1,2,3] Yi-Ju Ho,[1,2] Fabian Schmitt,[1,2] Dongyun Chen,[1,2] Johannes Kleinlein,[1,2] Wouter Beugeling,[1,2] Hartmut Buhmann,[1,2] Stanislau U. Piatrusha,[1,2] and Laurens W. Molenkamp[1,2,3]

[1]*Experimentelle Physik III, Physikalisches Institut, Universität Würzburg, Am Hubland, 97074 Würzburg, Germany.*

[2]*Institute for Topological Insulators, Universität Würzburg, Am Hubland, 97074 Würzburg, Germany*

[3]*Max Planck Institute for Chemical Physics of Solids, Nöthnitzer Straße 40, 01187 Dresden, Germany*

(Dated: November 17, 2025)

Supplementary Note 1. MAGNETORESISTANCE AT WEYL POINTS FOR DIFFERENT FIELD ORIENTATION

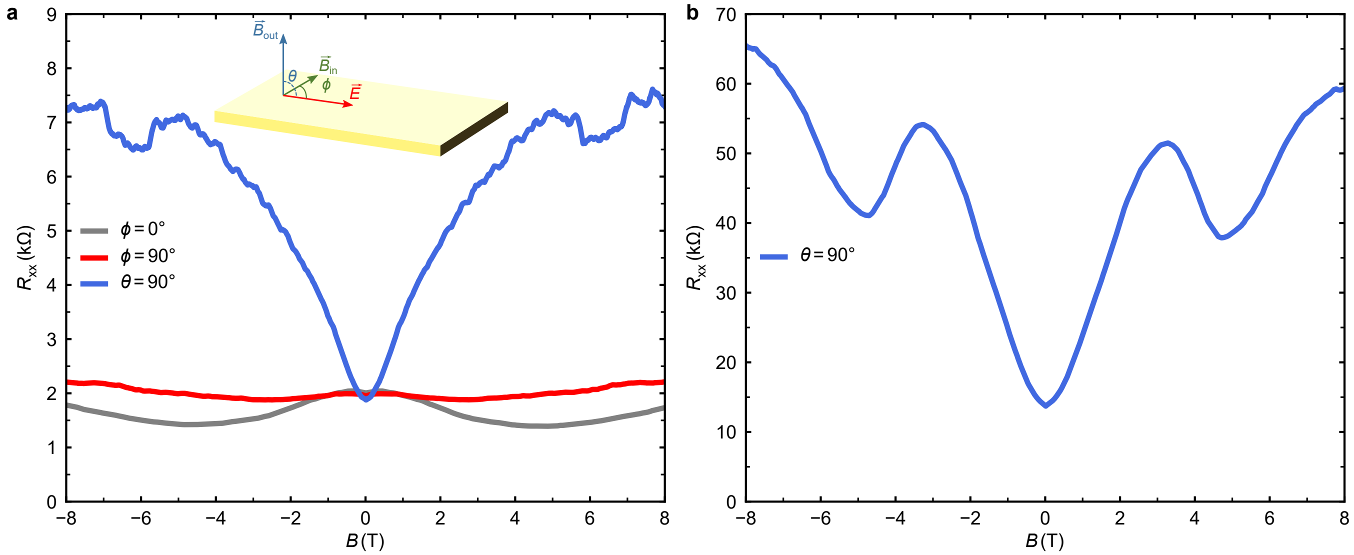

Supplementary Fig. 1. **a** Longitudinal magnetoresistance R_{xx} of the compressively strained HgTe island in the Weyl regime ($V_{g,i} = -0.29\,V$) for different angles between the magnetic field and the electric field. Angle θ defines the out-of-plane orientation of magnetic field, while ϕ is the in-plane angle between magnetic and electric fields (see schematic in the inset). **b** R_{xx} measured for one of the 40 µm-long channels in the Weyl regime for the out-of-plane field configuration.

Supplementary Fig. 1a shows the longitudinal magnetoresistance R_{xx} of the island, measured at $T_0 \approx 1.33\,K$ for different magnetic field orientations with respect to the direction of electric field. The zero-field R_{xx} values differ slightly, due to the variations in sample resistance after thermal cycling to room temperature. The strong negative magnetoresistance is exclusively observed for magnetic field direction parallel to the electric field (gray, $\phi = 0°$), while other in-plane configuration produces much weaker negative magnetoresistance (red, $\phi = 90°$). Out-of-plane magnetic field results in strong positive magnetoresistance (blue, $\theta = 90°$), with small oscillations for $|B| > 2\,T$. These oscillations are related to the finite-size ballistic transport effects in island, as they are not observed in R_{xx} of a larger, 40 µm-long channel in the Weyl regime, measured on the same device (see Supplementary Fig. 1b). There we observe a single oscillation at approximately 5 T, which corresponds to a transition between Landau levels, as previously reported in Ref. [1].

Supplementary Note 2. MEASUREMENT SETUP FOR JOHNSON NYQUIST NOISE

Supplementary Fig. 2 shows a schematic diagram of the measurement setup for Johnson-Nyquist noise. A custom-built low-noise, low-temperature HEMT amplifier (LTA) with the voltage gain $A_{\mathrm{LT}} \approx 5$ preamplifies the voltage noise from the sample, followed by NF SA-220F5 amplifier at room-temperature with the voltage gain $A_{\mathrm{RTA}} \approx 200$. An Anritsu MS2830A spectrum analyzer (SA) detects the signal power in a small band around the resonant frequency of the LC tank circuit connected between the sample and LTA, f_{res}. The sample is enclosed in a chamber with helium exchange gas at low pressure. This chamber and the LTA are immersed into the helium bath, the temperature of which is maintained at $T_0 \approx 1.33\,\mathrm{K}$ via evaporative cooling. The sample is cooled to T_0 via the exchange gas within the chamber, which is controlled via a separate thermometer, mounted directly on the sample holder. In this configuration, the sample can be independently heated up using

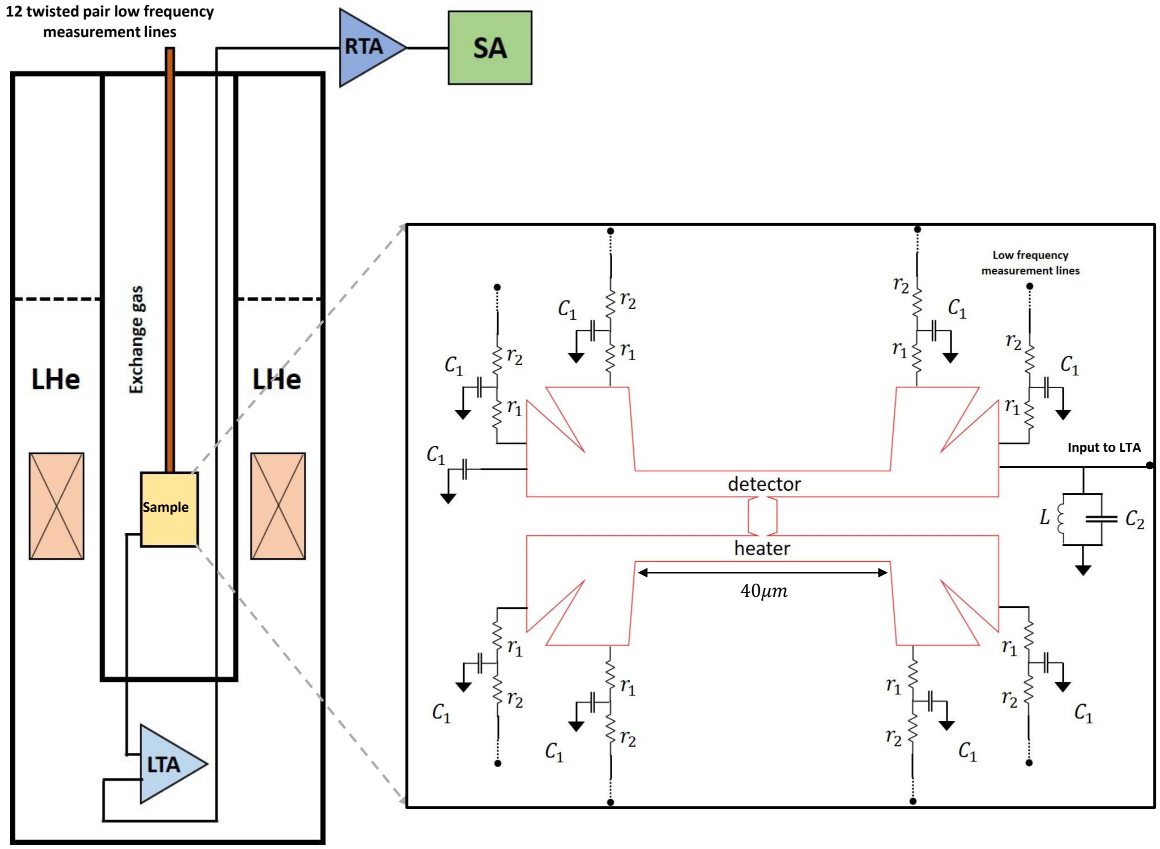

Supplementary Fig. 2. **The schematic for Johnson noise measurement setup.**

a dedicated heater, while the LTA temperature is kept constant at T_0.

The low-frequency lines, used for lock-in measurements, application of gate voltages, and heating currents, are fitted with RC low-pass filters that block the outside RF signals from reaching into the device. The resistance r_1 in these filters is selected to effectively choke the RF signals from the device, so that these lines have minimal influence on the high frequency noise measurements, while avoiding too high values, which could limit the frequency range of lock-in measurements. Based on our calculations, r_1 was set to 50 kΩ. This ensures that the LTA input is dominated by the voltage noise from the detector channel, while the pickup of voltage noise from the heater channel is attenuated to only 0.26% of its original value. The resistors r_2 (1 kΩ) and capacitors C_1 form low-pass filters with a cutoff frequency of 16 kHz that attenuate high frequency signals picked up via the measurement lines.

An extra capacitor with $C_1 = 10\,\mathrm{nF}$ is connected directly to one terminal of the detector channel and acts as a high-frequency ground, defining the measurement circuit for voltage noise. The opposite terminal of detector channel is connected to the LTA input via an approximately 50 cm long coaxial line. For impedance matching to the LTA input, a home-built coreless superconducting inductor with an inductance of $L = 10\,\mu\mathrm{H}$ is connected in parallel to the LTA input, so that it forms an LC tank circuit together with the parasitic capacitance of the coaxial line, $C_2 \approx 78\,\mathrm{pF}$. The noise measurements are performed around the resulting resonance frequency $f_{\mathrm{res}} \approx 5.7\,\mathrm{MHz}$.

Supplementary Note 3. IN-PLANE MAGNETORESISTANCE AND DIFFERENTIAL RESISTANCE OF HEATER AND DETECTOR CHANNELS

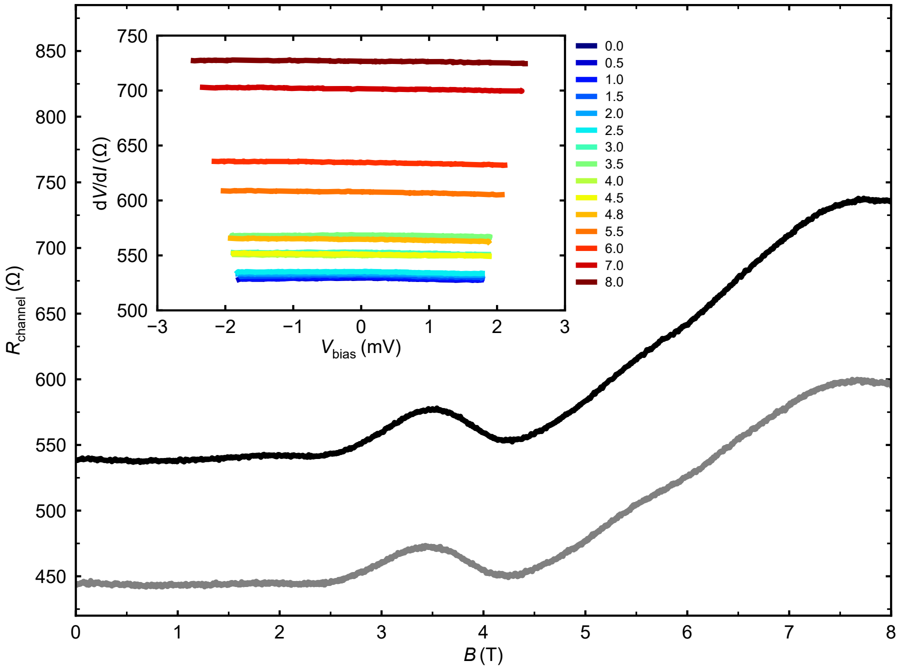

Supplementary Fig. 3. **In-plane magneto-resistance of the heater channel for its Fermi level tuned to the n-conducting regime (2D carrier density $n \approx 3{\times}10^{11}$cm^{-2}).** Black: two-terminal resistance (including resistance at the interfaces between HgTe layer and metal leads), gray: four-terminal resistance, measured using different contact pairs for current biasing and voltage measurement. The inset shows the differential two-terminal resistance of the heater for different in-plane B fields, ranging from 0 to 8T.

Supplementary Fig. 3 shows the in-plane magnetoresistance of the heating channel, measured for the Fermi level high in the conduction band ($n \approx 3{\times}10^{11}$cm^{-2}). In this measurement the electric field within the channel is oriented perpendicular to the in-plane magnetic field, which corresponds to the heating experiment with field parallel to the heat flow in the island. The channel demonstrates weak positive magnetoresistance, with only minimal variation up to 5 T. Beyond this point, an approximately 37% increase in magnetoresistance is recorded. We account for this change in channel resistance when extracting the electron temperatures at magnetic fields. Additionally, the inset of Fig. 3 shows the differential resistance dV/dI of the channel as a function of applied DC bias. We observe less than 0.5% variation for all

applied B-fields, which ensures that we always operate in the linear regime.

Supplementary Note 4. HEAT RELAXATION TO PHONONS FOR DIFFERENT TRANSPORT REGIMES

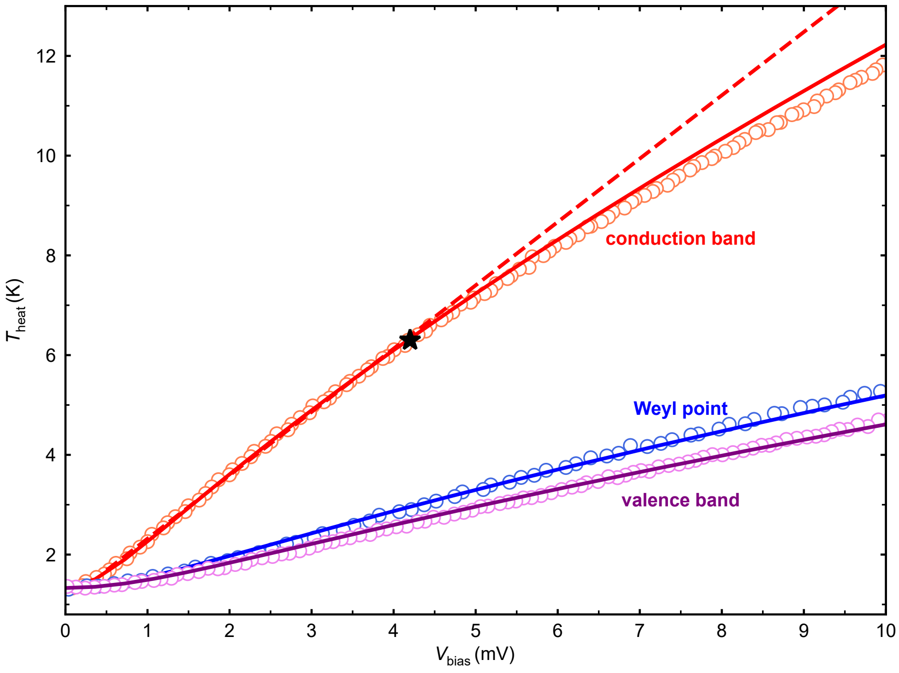

Supplementary Fig. 4. **Heat relaxation to the phonons at larger DC bias voltage.** Excitation dependent electron temperature in the heater channel, $T_{\rm heat}$ for different Fermi level positions: conductance band (red), Weyl point (blue) and valence band (violet). Solid lines corresponds to the phonon relaxation model while circles represents the experimentally measured data.

In Supplementary Fig. 4, we show the study the electron-phonon relaxation in the channel. We measure the average heater temperature $T_{\rm heat}$ while applying large heating current at $B = 0\,{\rm T}$. The measurement is performed for three different positions of the Fermi level in the channel: in the conduction band (which used in all other thermal conductance experiments), close to the Weyl points, and the valence band, so that electric transport is happening via the holes. In the conduction band (orange symbols), we clearly observe a characteristic bending away from the linear trend (dashed line), starting at a position indicated by a black star, suggesting increasing phonon contribution to heat relaxation at larger temperatures. For the other two regimes (blue and violet), the heating at the same bias results in much lower temperature, suggesting that phonon contribution is notable from small heating biases.

We evaluate the electron-phonon (or hole-phonon) relaxation by simulating $T_{\rm heat}$ using the

finite element model based on heat transport equation, incorporating the heat relaxation in the form $q_{\mathrm{ph}} = \Sigma_{\mathrm{ph}}(T^3 - T_0^3)$ and taking into account the channel resistance in the corresponding regime (solid lines). We obtain the best match for the identical coefficient Σ_{ph}=0.19 Wm^{-2}K^{-3} in the electron regime and at the Weyl point, while for the Fermi level in the valence band Σ_{ph}=0.36 Wm^{-2}K^{-3} is approximately double, indicating enhanced hole-phonon scattering, likely due to the larger effective mass of the holes. In all three regimes $T^3 - T_0^3$ results in the best fit compared to other possible powers.

Supplementary Note 5. DEMONSTRATION OF IDENTICAL HEAT RELAXATION IN HEATER AND DETECTOR CHANNELS

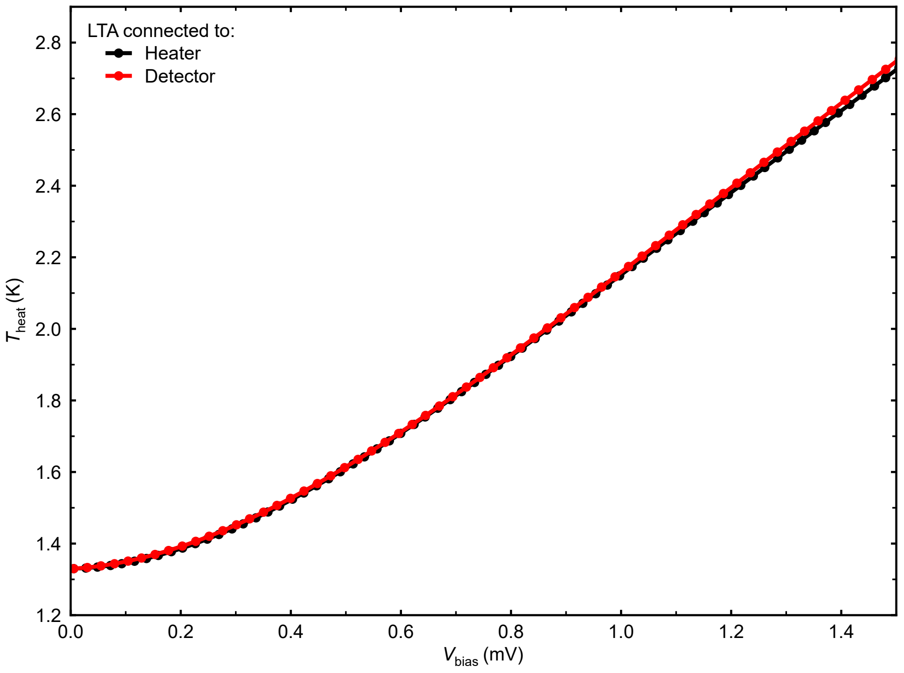

Supplementary Fig. 5. **Comparison between the temperature response of heater and detector channels.** Electronic temperature in the heater channel (black) and the detector channel (red) in response to the voltage bias $V_{\text{bias}} = I_{\text{heat}} R_{\text{ch}}$. In both measurements the heating current was applied to the same channel, on which the noise measurement was performed. The close match between the curves confirms the assumption of identical heat relaxation mechanisms in the H-bar channels.

In the main experiment, Johnson noise thermometry was performed on one of the two channels, referred to as the "detector." When a heating current I_{heat} was applied through this same channel, we label the measured electron temperature as T_{heat}. When the current is applied through the opposite channel, we label the measured temperature as T_{det}. Our analysis relies on the assumption that the two channels have identical thermal transport properties, which is supported by their closely matching resistances R_{ch}.

To further validate that the heat relaxation mechanisms are indeed equivalent in both channels, we performed a complementary measurement with the low-temperature amplifier (LTA) connected to the opposite channel. This required a thermal cycle to reconfigure the

wiring but caused no significant change in the gate dependence of the channel resistances.

Figure 1 shows the electron temperature response as a function of I_{heat} for both configurations: one used for the data in the main text (red), and the other with the LTA connected to the opposite channel (black). The near-identical temperature responses confirm that the two channels exhibit matching thermal properties, justifying our use of them interchangeably in the analysis.

Supplementary Note 6. SPATIAL TEMPERATURE PROFILES IN THE HEATER AND DETECTOR CHANNELS

The temperature profiles in the heater and detector channels were simulated using finite element method, accurately adapting the device geometry shown in the inset of Supplementary Fig. 6d and taking into account the determined electron-phonon relaxation rate. Supplementary Fig. 6a and Supplementary Fig. 6b depict the temperature profiles along the center of the heater (black) and detector (red) channels under different heating excitations of 200 µV and 2 mV, respectively.

The heater channel exhibits a temperature profile reminiscent of a parabola due to the uniform Joule heating, while the detector channel has a more linear profile, as the heat is injected in the middle of the channel [2]. Notably, large contact areas at the channel ends have local temperature change with coordinate slower, than in the main narrow strip. The temperature profiles in both the heater and detector channel are affected by the magnitude of heating (Fig. 6b).

Supplementary Fig. 6c illustrates the correlation between the average noise temperature of the heater (T_{heat}) and the detector (T_{det}) channels and corresponding local temperatures close to the island entrance (T_{hot} for the heater and T_{cold} for the detector). The average temperatures were calculated via integrating the local temperatures weighted according to their contribution to the total noise.

From our model we determine the conversion functions of T_{heat} into T_{hot} and T_{det} into T_{cold}, and use them for finding the local temperatures from our measurements. We find, that despite the geometry correction and phonon contribution the relationship between T_{heat} and T_{hot} is close to the one predicted from the analytical solution (Eq. 3 in the main text), while for the detector channel $T_{\mathrm{cold}}/T_0 - 1 \approx 2.63 \cdot (T_{\mathrm{det}}/T_0 - 1)$ with a factor 2.63 differing from 2,

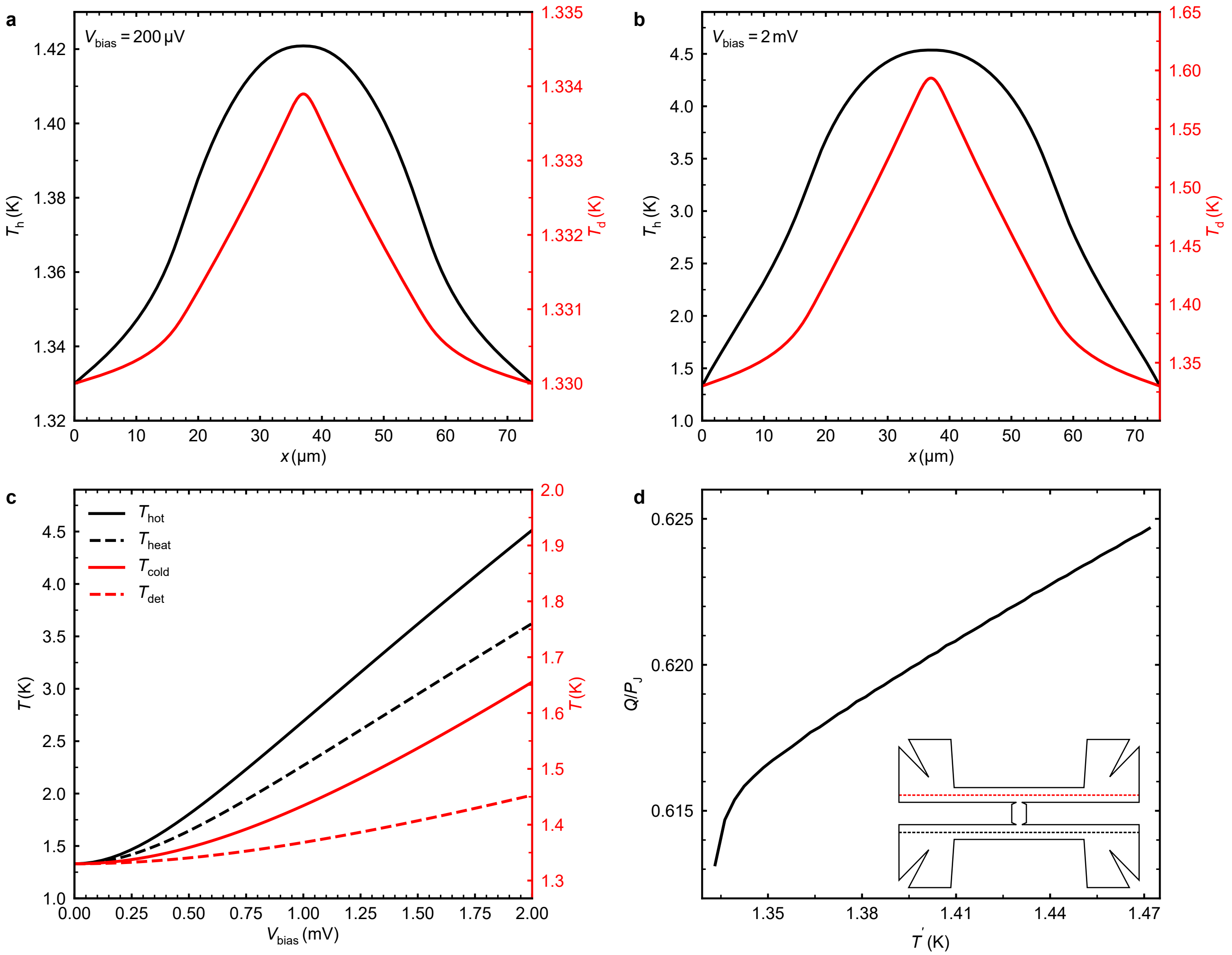

Supplementary Fig. 6. Modeling of the thermal transport in heater and detector channels. **a** Temperature profiles along the heater and detector channels for the DC bias $V_{\rm bias} = 200\,\mu$V applied to the heater. The local temperatures are extracted along the line going through the middle of the channel strip (see inset in panel **(d)**). **b** The same as **(a)**, but for larger bias $V_{\rm bias} = 2\,$mV. **c** Average channel temperatures ($T_{\rm heat}$ and $T_{\rm det}$) and local temperatures close to the island entrance and exit ($T_{\rm hot}$ and $T_{\rm cold}$) for different heating bias. **d** Temperature-dependent ratio of heat flow across the island to the dissipated heating power in the channel, $Q/P_{\rm J}$. The inset illustrates the layout of the sample design used for our model. The black and red dashed line corresponds to the positions where temperature profile was extracted for panels **(a)** and **(b)** for heater and detector respectively.

expected for purely diffusive heat transport. An identical analysis was conducted for data

at each magnetic field.

The slight modifications in the temperature profiles of the heater and detector are also reflected in the small change in the ratio of heat flow across the island to the dissipated heating power in the channel, Q/P_J. This ratio is also dependent on the heating excitation and was found to deviate approx. 10% from the 2/3, expected for a pure diffusive limit (Fig. 6d).

Our approach to calibrating the heat flow Q via P_J is reliable, as most of the heat, produced in the heater channel, is dissipated directly on the heater side, and only a small fraction is transported to the detector as Q. This is obvious from Fig. 3a in the main text, where we observe that the temperature increase $T_\mathrm{heat} - T_0$ is more than ten times larger than $T_\mathrm{det} - T_0$. When Q is small, the temperature profile in the heater channel should not be affected much by the heat flow into the island.

Supplementary Note 7. ERROR ANALYSIS FOR THE THERMAL CONDUCTANCE MEASUREMENT

Both the X and Y axes in Fig. 3b, where $X = (T_\mathrm{hot}^2 - T_\mathrm{cold}^2)$ and $Y = Q \approx \alpha_\mathrm{Q} P_\mathrm{J}(T_\mathrm{heat} = T_\mathrm{det})$, contribute to the uncertainty in the thermal conductance, κ. The corresponding standard deviations δX_i and δY_i are calculated from the experimental standard deviations of T_hot and T_cold for each point (indexed with i):

$$\delta X_i = \sqrt{\left(\frac{\partial X}{\partial T_\mathrm{hot}}\right)^2 (\delta T_\mathrm{hot})^2 + \left(\frac{\partial X}{\partial T_\mathrm{cold}}\right)^2 (\delta T_\mathrm{cold})^2} = 2\sqrt{T_\mathrm{hot}^2 (\delta T_\mathrm{hot})^2 + T_\mathrm{cold}^2 (\delta T_\mathrm{cold})^2}$$

$$\delta Y_i = \sqrt{\left(\frac{\partial Y}{\partial T_\mathrm{heat}}\right)^2 (\delta T_\mathrm{heat})^2 + \left(\frac{\partial Y}{\partial T_\mathrm{det}}\right)^2 (\delta T_\mathrm{det})^2}$$

The standard deviation $\delta\kappa$ is estimated for weighted linear regression:

$\delta\kappa = \sqrt{\frac{1}{S_{xx}}}$ where $S_{xx} = \sum_{i=1}^{n} w_i X_i^2$ and the effective weight, $w_i = \frac{1}{\delta Y_i^2 + \kappa^2 \delta X_i^2}$ The error bars in the main graph represent the 95% confidence interval based on $\delta\kappa$.

Supplementary Note 8. CARRIER DENSITY AND MOBILITY MEASUREMENTS

Carrier density and mobility as a function of gate voltage were measured at 4.2 K on a separate gated Hall-bar device fabricated from the same wafer (internal reference QC0739). The device has a standard eight-terminal geometry with a total length of 1200 µm and width of 200 µm. The longitudinal resistance R_{xx} was recorded between the nearest voltage probes, giving an effective aspect ratio of 3.

In the n-conductance regime, the carrier density was extracted from the slope of the classical Hall effect in R_{xy}, and the mobility from the zero-field resistance. In the p-regime, both the net charge carrier density (i.e. electron minus hole density) and mobility were obtained from a two-carrier analysis of R_{xx} and R_{xy} at small fields. Supplementary Table S1 summarizes the extracted net carrier densities corresponding to the island gate voltage $V_{g,i}$ used in the main text. These values are offset by -0.3 V relative to the Hall-bar gate voltage $V_{g,hall}$.

TABLE S1. Extracted carrier density and mobility for the Hall bar device.

$V_{g,i}$ (V)	$V_{g,hall}$ (V)	n (10^{11} cm^{-2})	Mobility μ (10^3 cm^2/[Vs])
0.3	0.6	3.74	77.6
0.1	0.4	3.20	62.2
-0.1	0.2	2.27	35.5
-0.15	0.15	1.96	28.5
-0.2	0.1	1.61	22.8
-0.4	-0.1	-1.1	13.3
-0.5	-0.2	-2.24	15.7
-0.6	-0.3	-2.84	18.5

Supplementary Fig. 7 shows for the Hall bar device the R_{xx} as a function of in-plane magnetic field in the direction of current transport $B_{\parallel}$ and out of plane field $B_{\perp}$, Hall effect in R_{xy} as a function of $B_{\perp}$, and $V_{g,hall}$ dependence of zero-field resistance, showing exact match with the data from H-bar from the main text.

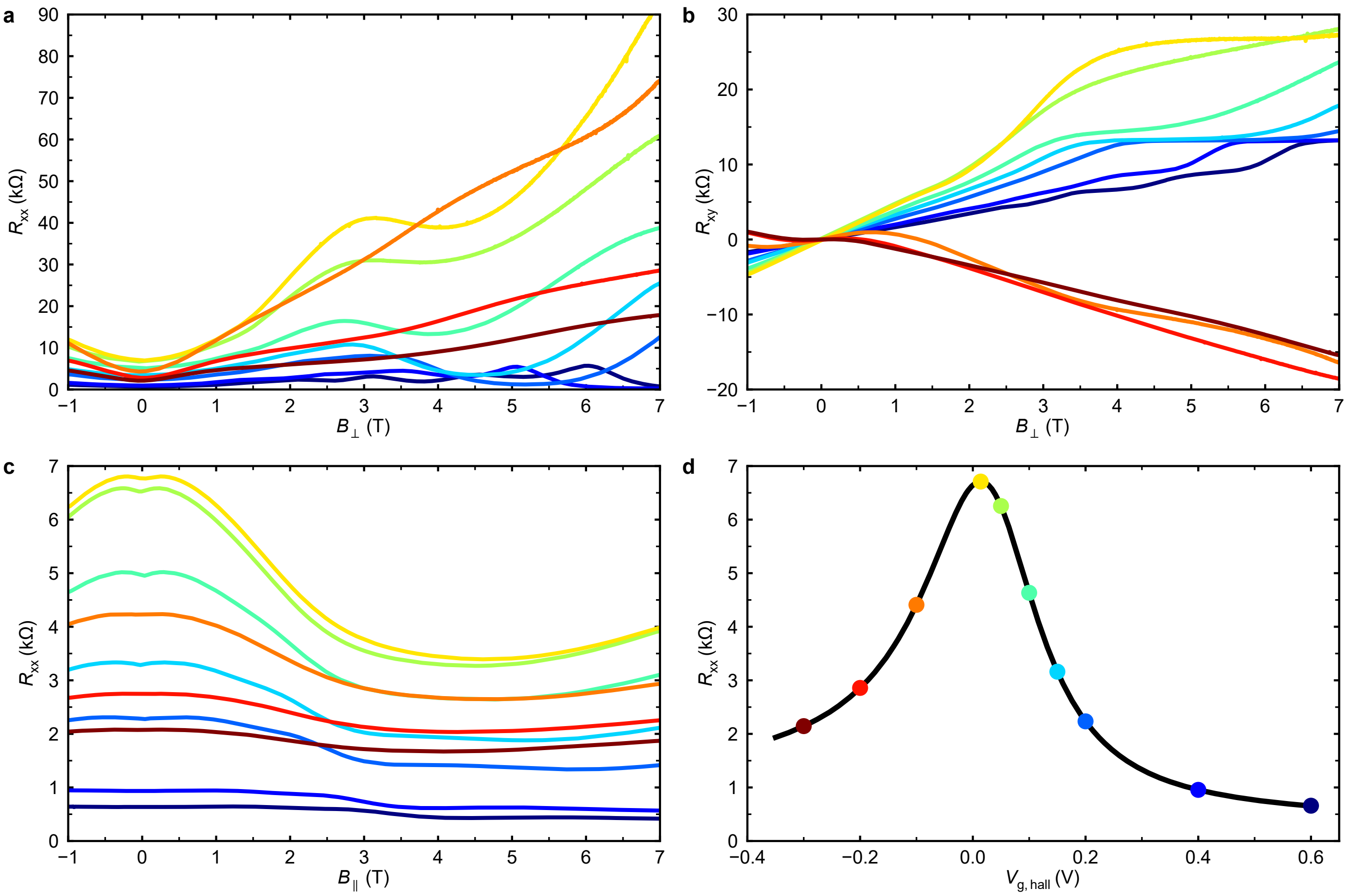

Supplementary Fig. 7. **Magnetoresistance measurements on a** 1200 μm × 200 μm **Hall bar device. a** Longitudinal magnetoresistance R_{xx} as a function of out-of-plane magnetic field $B_\perp$. Measurements are performed at Fermi level positions, marked as the colored dots in panel **(d)**. **b** Transverse magnetoresistance R_{xy} measured alongside the data in panel **(a)**. **c** R_{xx} as a function of in-plane magnetic field. **d** R_{xx} at zero field as a function of Hall bar gate voltage $V_{\mathrm{g,hall}}$.

---

[1] D. M. Mahler, J.-B. Mayer, P. Leubner, L. Lunczer, D. Di Sante, G. Sangiovanni, R. Thomale, E. M. Hankiewicz, H. Buhmann, C. Gould, and L. W. Molenkamp, Interplay of Dirac nodes and Volkov-Pankratov surface states in compressively strained HgTe, Phys. Rev. X 9, 031034 (2019).

[2] C. Pozderac and B. Skinner, Relation between Johnson noise and heating power in a two-terminal conductor, Phys. Rev. B 104, L161403 (2021).
